# Hemangioblastoma and von Hippel-Lindau disease: genetic background, spectrum of disease, and neurosurgical treatment

**DOI:** 10.1007/s00381-020-04712-5

**Published:** 2020-06-07

**Authors:** Jan-Helge Klingler, Sven Gläsker, Birke Bausch, Horst Urbach, Tobias Krauss, Cordula A. Jilg, Christine Steiert, Alexander Puzik, Elke Neumann-Haefelin, Fruzsina Kotsis, Hansjürgen Agostini, Hartmut P.H. Neumann, Jürgen Beck

**Affiliations:** 1 Department of Neurosurgery, Medical Center - University of Freiburg, Faculty of Medicine, University of Freiburg, Breisacher Str. 64, 79106 Freiburg, Germany; 2 Department of Neurosurgery, Universitair Ziekenhuis Brussel, VUB University, Brussels, Belgium; 3 Department of Medicine II, Medical Center - University of Freiburg, Faculty of Medicine, University of Freiburg, Freiburg, Germany; 4 Department of Neuroradiology, Medical Center - University of Freiburg, Faculty of Medicine, University of Freiburg, Freiburg, Germany; 5 Department of Radiology, Medical Center - University of Freiburg, Faculty of Medicine, University of Freiburg, Freiburg, Germany; 6 Department of Urology, Medical Center - University of Freiburg, Faculty of Medicine, University of Freiburg, Freiburg, Germany; 7 Department of Pediatric Hematology and Oncology, Medical Center - University of Freiburg, Faculty of Medicine, University of Freiburg, Freiburg, Germany; 8 Department of Medicine IV, Medical Center - University of Freiburg, Faculty of Medicine, University of Freiburg, Freiburg, Germany; 9 Eye Center, Medical Center - University of Freiburg, Faculty of Medicine, University of Freiburg, Freiburg, Germany; 10 Section for Preventive Medicine, Medical Center - University of Freiburg, Faculty of Medicine, University of Freiburg, Freiburg, Germany

**Keywords:** Adolescence, Childhood, Hemangioblastoma, Screening, Surgery, VHL, von Hippel-Lindau

## Abstract

**Introduction:**

Hemangioblastomas are rare, histologically benign, highly vascularized tumors of the brain, the spinal cord, and the retina, occurring sporadically or associated with the autosomal dominant inherited von Hippel-Lindau (VHL) disease. Children or adults with VHL disease have one of > 300 known germline mutations of the *VHL* gene located on chromosome 3. They are prone to develop hemangioblastomas, extremely rarely starting at age 6, rarely at age 12–18, and, typically and almost all, as adults. There is a plethora of VHL-associated tumors and cysts, mainly in the kidney, pancreas, adrenals, reproductive organs, and central nervous system. Due to a lack of causal treatment, alleviation of symptoms and prevention of permanent neurological deficits as well as malignant transformation are the main task. Paucity of data and the nonlinear course of tumor progression make management of pediatric VHL patients with hemangioblastomas challenging.

**Methods:**

The Freiburg surveillance protocol was developed by combining data from the literature and our experience of examinations of > 300 VHL patients per year at our university VHL center.

**Results:**

Key recommendations are to start screening of patients at risk by funduscopy with dilated pupils for retinal tumors with admission to school and with MRI of the brain and spinal cord at age 14, then continue biannually until age 18, with emergency MRI in case of neurological symptoms. Indication for surgery remains personalized and should be approved by an experienced VHL board, but we regard neurological symptoms, rapid tumor growth, or critically large tumor/cyst sizes as the key indications to remove hemangioblastomas. Since repeated surgery on hemangioblastomas in VHL patients is not rare, modern neurosurgical techniques should encompass microsurgery, neuronavigation, intraoperative neuromonitoring, fluorescein dye-based intraoperative angiography, intraoperative ultrasound, and minimally invasive approaches, preceded in selected cases by endovascular embolization. Highly specialized neurosurgeons are able to achieve a very low risk of permanent morbidity for the removal of hemangioblastomas from the cerebellum and spinal cord. Small retinal tumors of the peripheral retina can be treated by laser coagulation, larger tumors by cryocoagulation or brachytherapy.

**Conclusion:**

We consider management at experienced VHL centers mandatory and careful surveillance and monitoring of asymptomatic lesions are required to prevent unnecessary operations and minimize morbidity.

## Introduction

Hemangioblastoma of the central nervous system (CNS) occurs as a sporadic tumor or as a component of the hereditary von Hippel-Lindau (VHL) disease. The classical report dates back to 1926 when the Swedish pathologist Arvid Lindau (1892-1958) presented his thesis on cystic cerebellar tumors [1]. Lindau studied these tumors at the Seraphimer Hospital in Stockholm and extended his research by a longer journey to Berlin, Leipzig, Prague, and other university institutes where he saw autopsy protocols and specimen of patients with additional visceral tumors of the kidney, pancreas, and adrenals [2]. Comparing the histology of cerebellar tumors and those of the eyes in several of such patients, he provided evidence that both are identical. Today we use the term angioma or hemangioblastoma of the retina. It was the eminent neurosurgeon Harvey Cushing who suggested naming the disorder Lindau’s disease. The modest Swede, however, underscored the impact of the German ophthalmologist Eugen von Hippel (1867-1939), and now both names are used equivocally.

## Definitions, histology, and pathogenesis of hemangioblastomas and of VHL disease

**Fig. 1:**
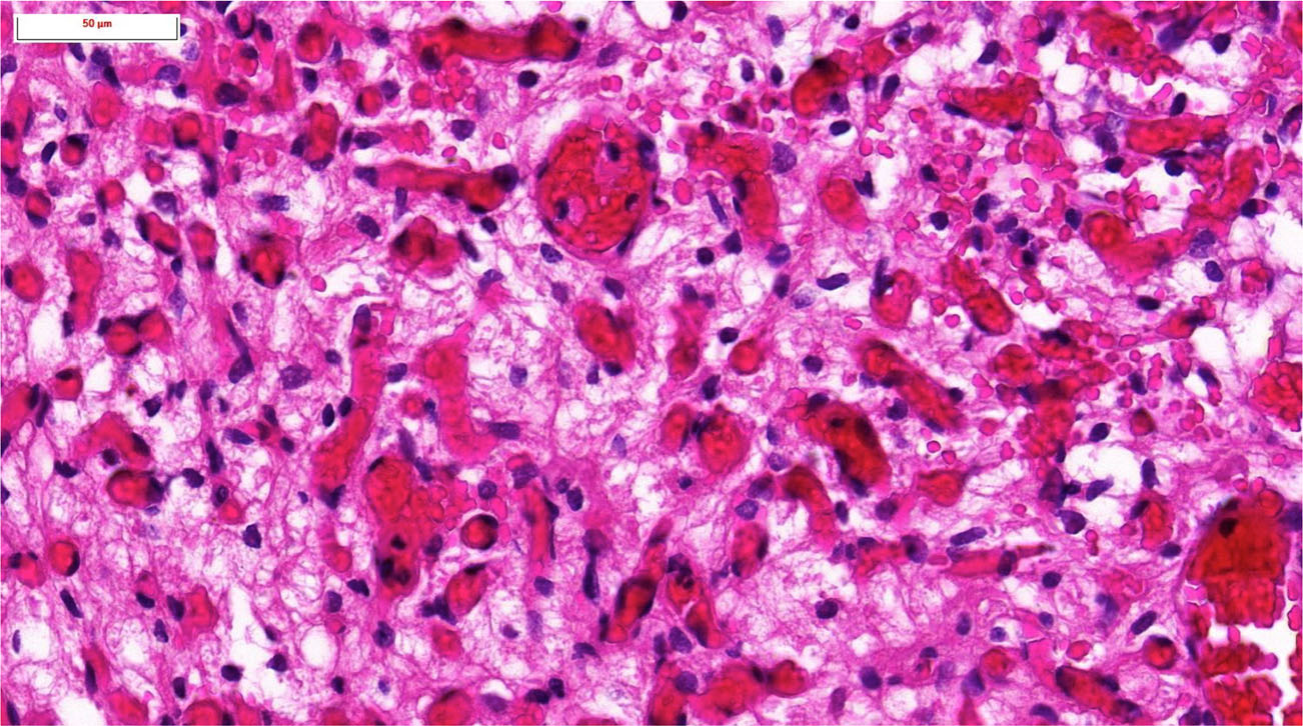
Histology of a hemangioblastoma of the central nervous system showing foamy stromal cells and abundant vessels

Hemangioblastomas are benign tumors of the CNS and are defined by their histopathology [3]. Striking components are lipid-loaden foamy cells and abundant capillaries (Fig. 1). In addition, many hemangioblastomas of the CNS form cysts of various sizes. The cysts are filled by amber-colored fluid. The main component of this fluid is erythropoietin. VHL is a syndrome that is characterized by various tumors and cystic lesions in several organs, as presented in detail below. Clinically, two presentations are observed: (i) an individual with a family history of VHL and the presence of a CNS or retinal hemangioblastoma, a pheochromocytoma or a clear cell renal cell carcinoma; or (ii) an index case (an individual with no family history) with two or more hemangioblastomas or two or more visceral tumors or one hemangioblastoma and one visceral tumor [4]. Today the diagnosis is mostly based on molecular findings. One lesion and the presence of a pathogenic *VHL* germline mutation lead to diagnosis.

Biallelic inactivation of the *VHL* tumor suppressor gene is thought to initiate tumorigenesis. The first allele is inactivated by a germline mutation or deletion and the second allele by deletion or mutation at a later stage. Inactivation of the *VHL* tumor suppressor gene has several consequences on the molecular level. The most prominent is dysregulation of oxygen sensation with upregulation of hypoxia inducible factor (HIF) and its several downstream targets. The Nobel Prize for Medicine and Physiology 2019 was awarded to the researchers who discovered this mechanism. HIF induces angiogenesis, blood formation, metabolic changes, and several other effects. In addition, *VHL* tumor suppressor gene inactivation has also several HIF-independent effects including cell cycle regulation and apoptosis.

*VHL* germline mutations cause a variety of developmental changes and tumors in humans. Although the germline mutations are present in every cell of the body, the disease targets a highly selective subset of organs including the nervous system, eye, inner ear, kidney, pancreas, adrenal gland, and epididymis or broad ligament. The affected organs reveal numerous microscopic developmental changes and pre-neoplastic lesions. From these lesions, tumorigenesis may occur later on. In analogy in all affected organs, the tumors develop in a stepwise progression from precursor lesions to different stages of tumor growth [5–7].

In the CNS, the microscopic precursor lesions occur mainly in the dorsal roots [8] and in the molecular layer of the cerebellum [9]. The cells of origin are probably developmentally arrested hemangioblast precursor cells [5, 10]. Interestingly, the microscopic precursors already reveal biallelic *VHL* inactivation even if they are not growing. Initiated by mechanisms, which are not yet known, these microscopic cell clusters begin to cause reactive angiogenesis. A small subset of clusters may then grow further. They grow from the dorsal roots into the spinal cord without infiltrating it. The cells start to increase their cytoplasm revealing a foamy appearance. The capillary network becomes dilated and the tumors look similar to clear cell renal carcinoma [11]. Next to the neoplastic “stromal cells,” the other cytologic component of hemangioblastomas is represented by abundant blood vessels. The blood vessels do not show *VHL* inactivation [12, 13] and therefore represent reactive angiogenesis. During progression, the hemangioblastoma tumor cells imitate the morphology of the hemangioblast, an embryonic precursor cell for blood, endothelium, and smooth muscle. Larger and fast-growing hemangioblastomas typically reveal features of this more differentiated stage [11]. In progressed stages, hemangioblastoma tumor cells can reveal blood island formation and extramedullary hematopoiesis. They can also differentiate into vasculogenetic structures [14].

## Prevalence

Hemangioblastoma accounts for approximately 0.5% of all tumors of the CNS. For the spinal tumors, hemangioblastoma accounts for 2.1% of all spinal tumors in the SEER (Surveillance, Epidemiology, and End Results) database of the NIH [15].

CNS hemangioblastomas are seen in about 60–80% of VHL patients, with a remarkable difference within and among affected families [16, 17]. In some, kindred CNS hemangioblastomas are the dominant lesion and present in up to all patients, whereas in others, penetrance for these tumors is low or even zero.

## VHL-associated tumors

The spectrum of manifestations of the VHL disease comprises developmental changes with multiple pre-neoplastic lesions as well as cysts and tumors in various organs. Of these tumors, some are highly prone to become malignant and to settle local or distant metastases, in particular, clear cell renal cell carcinomas and pancreatic neuroendocrine tumors [18, 19]. The risk for metastases of kidney and pancreas tumors is dependent on size (odds ratio 1.25 for each 1 cm increase, *p* < 0.001) [20]. We recommend a cut-off diameter for renal surgery of 4 cm for renal cell cancer and 2.5 cm for pancreatic neuroendocrine tumors [19–23]. The decision for nephron sparing surgery should be done extremely carefully because recurrent or new renal tumors will occur and recurrent renal surgery is inevitable. In contrast, retinal hemangioblastomas are always histologically benign neoplasms similar to their CNS counterparts. Their impact is the risk for uni- or bilateral blindness due to detachment of the retina or if they are located at the optic disc where treatment options like proton beam radiation are limited and always associated with loss of vision. Intermedian tumor entities are pheochromocytomas and paragangliomas; in these, metastases occur in about 5% of the patients. Other components of the VHL disease are endolymphatic tumors of the inner ear (ELST) and cysts or cystadenomas of the epididymis and the broad ligament. Their clinical relevance is less prominent, but uni- or bilateral deafness can occur, and infertility has been described in men. Characteristic for the visceral VHL-associated tumors is a multifocal and bilateral, simultaneous, or metachronous occurrence.

## Genetics

von Hippel-Lindau disease is an autosomal dominant disorder with high, but age-dependent penetrance. Underlying are germline mutations of the *VHL* gene located on chromosome 3p25-26 encoding the VHL protein of 213 amino acids [24]. The mutations are randomly distributed over the entire gene. Missense or truncating mutations as well as deletions and rearrangements of the entire gene occur. Truncating mutations comprise nonsense mutations, intraexonic deletions, and insertions of one or more nucleotides and splice site mutations. Patients with truncating mutations are often more severely affected compared with those with missense mutations. Hot spots of mutations are the nucleotides 499 and 500 in codon 167.

Screening procedure for the molecular diagnosis of VHL was initially Sanger sequencing of the 3 exons of the *VHL* gene [25]. For the detection of large deletions, MLPA is the method of choice [26]. Recently, next-generation sequencing including the whole exon/whole genome has been introduced as a highly diagnostic procedure [27].

## Age at manifestation

The detection of CNS hemangioblastomas can be distributed across the 1st to the 8th decade of life. The majority of VHL patients are diagnosed in their thirties and forties. Sporadic, single hemangioblastoma is diagnosed about 10–20 years later compared with its syndromic counterpart. Both sporadic and VHL-associated hemangioblastomas are extremely rare in childhood (incidence < 1 per 1,000,000). The youngest age of VHL-associated CNS hemangioblastoma is reported at 6 years [28]. But the most common early manifestations in VHL patients < 18 years are retinal (34%) and CNS (29%) hemangioblastoma [28]. If a CNS hemangioblastoma is first detected in childhood, an underlying VHL disease should always be suspected and further examined [29, 30].

## Symptoms and signs

CNS hemangioblastomas cause various symptoms depending on the tumor size and localization. Typically, most of the space-occupying process is caused by the cystic component of the tumor [31]. These tumor-associated cysts occur more frequently in younger individuals and seem to progress more rapidly [32].

Cerebellar tumors can be associated with headache, gait imbalance, ataxia, and abnormal head position. Large cerebellar tumors can cause nausea, vomiting, and papilledema. Brainstem and spinal hemangioblastomas may show radix-specific neurological deteriorations, urinary or bowel abnormalities, singultus, dysphagia, myelopathic disorders, or symptoms of syringomyelia. Some hemangioblastomas are associated with polyglobulia [33–35]. Hemorrhage from the highly vascularized CNS hemangioblastoma is very rare, but has been reported [36]. The risk of hemorrhage is very low; it was calculated at 0.0024 per person per year and predominantly occurs with large tumors [37].

Compared with adults, pediatric VHL patients have a higher prevalence of obstructive hydrocephalus and tumor-associated cysts. This is associated with a shorter duration from onset of symptoms to surgery of 1.5 ± 2.1 months (range, 3 days–12 months) [29] compared with adult VHL patients with a duration of 8 months [38]. It is important to note that CNS hemangioblastoma in children might remain clinically occult until severe symptoms occur. The same applies for retinal hemangioblastoma.

## Radiology, locations of hemangioblastomas

**Fig. 2:**
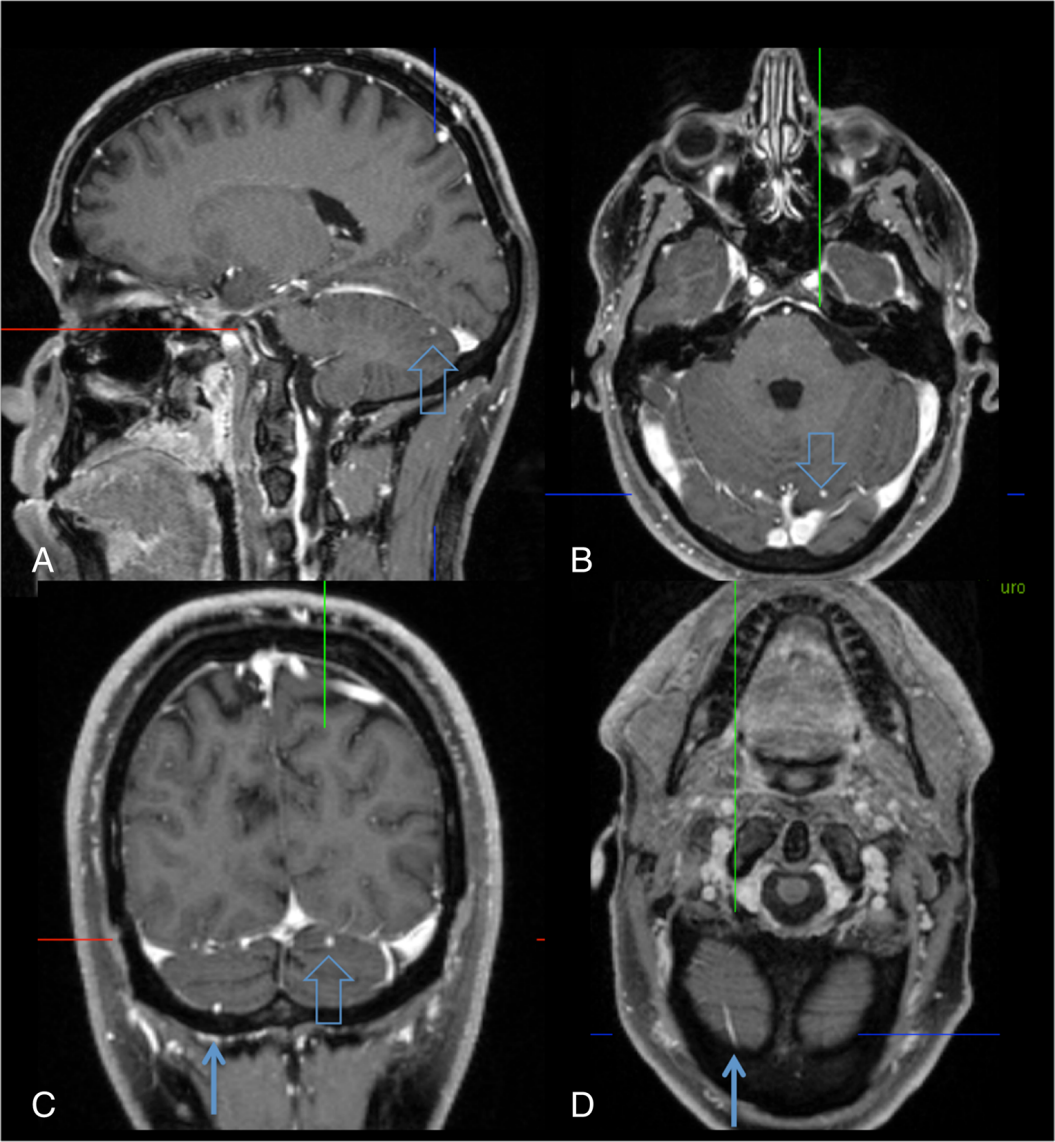
Tiny hemangioblastoma in the left cerebellar hemisphere in a patient with VHL disease (**a**–**c**: hollow arrow). In order to distinguish this tiny lesion from a vessel, high-resolution images allowing multiplanar reformations are needed. An example is the vessel (**c**, **d**: arrow), which in axial reformation is displayed as a tubular structure

A total of 60–80% of VHL patients are considered to have cerebellar and 40–59% spinal, often multiple hemangioblastomas. However, the prevalence may be even higher considering MRI scans that show multiple tiny nodules visible on high-resolution T1-weighted contrast-enhanced sequences (Fig. 2).

**Fig. 3:**
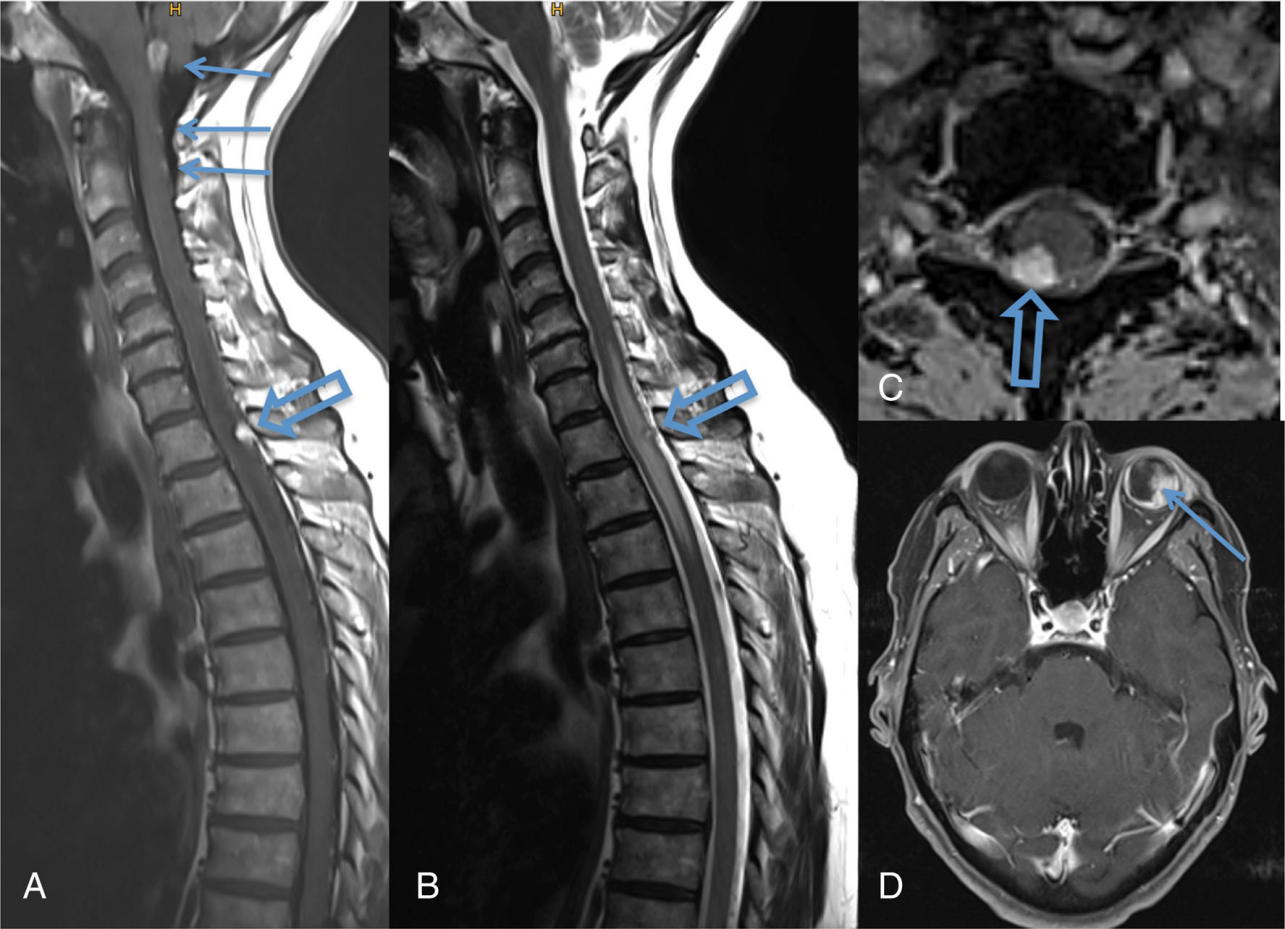
Patient with VHL disease and multiple hemangioblastomas in the obex and the dorsal aspect of the spinal cord. All lesions have a pial contact. The one at level Th1 (**a**–**c**: hollow arrow) has caused a spinal cord edema. Axial T1-weighted contrast-enhanced images with fat sat also show a left-sided retinal “angioma” (**d**: arrow)

**Fig. 4:**
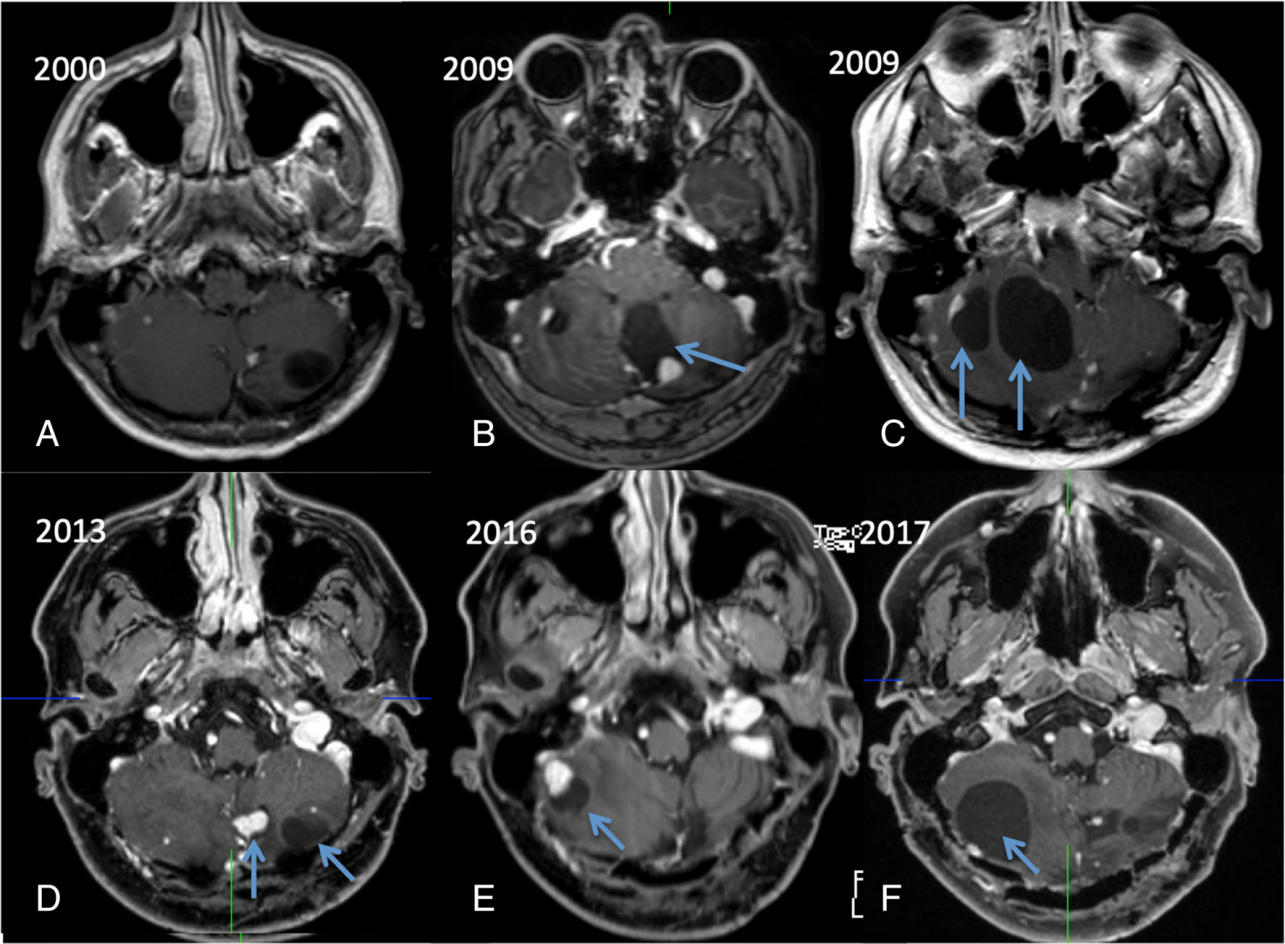
Multiple cerebellar hemangioblastomas remained stable for several years (**a**). In 1 year, especially cystic portions in the left inferior vermis (**b**: arrow) and afterwards in the right hemisphere got larger (**c**: arrow). Some years later, solid and cystic (**d**, **e**: arrows) and cystic hemangioblastoma portions (**f**: arrow) started to grow

**Fig. 5:**
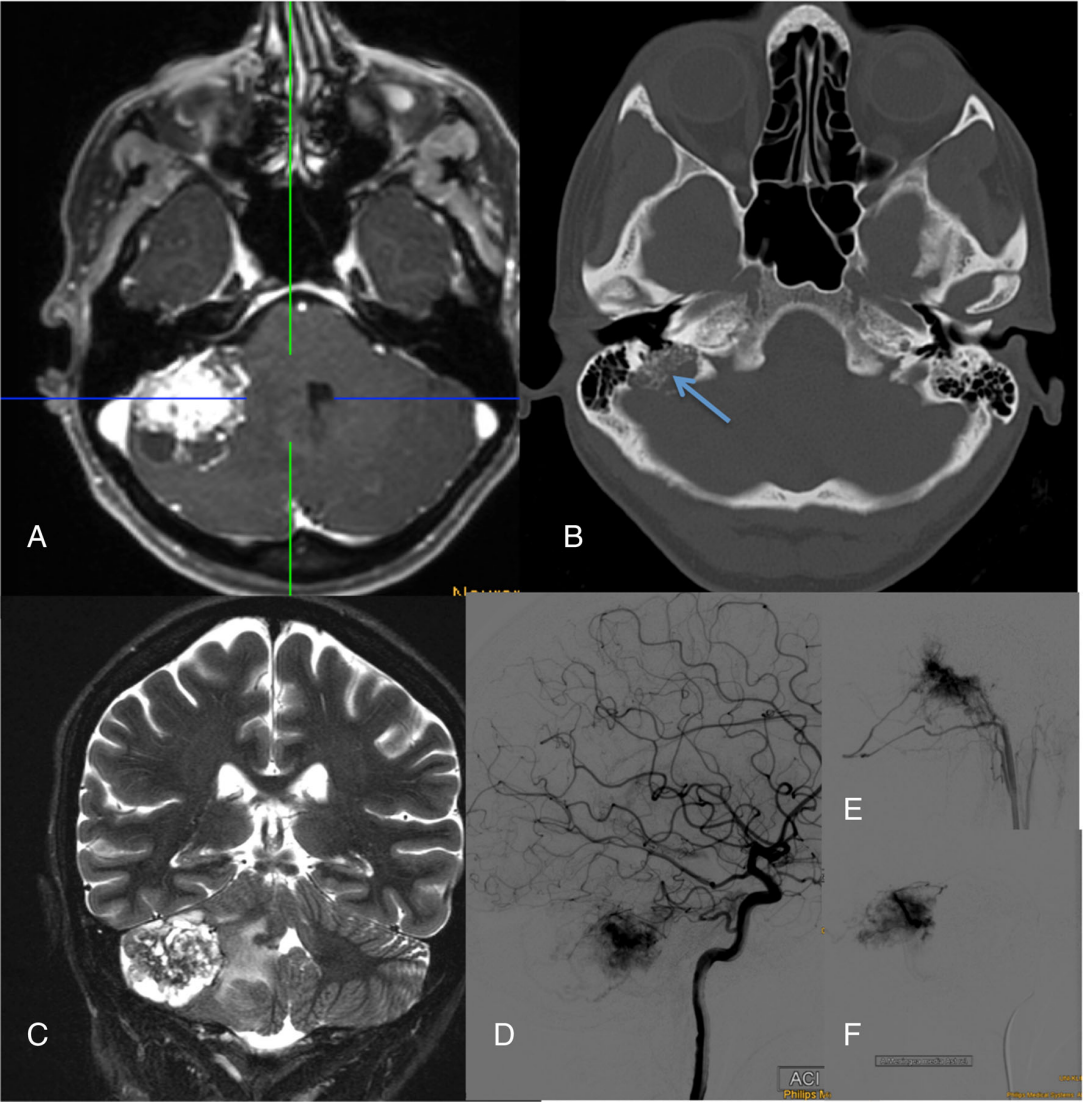
Endolymphatic sac tumor (ELST) in a typical location with strong enhancement (**a**), calcific spiculae on CT (**b**: arrow), inhomogeneous signal due to calcifications and cysts on T2-weighted images (**c**), and intense vascular blush with supply via branches of the ICA (**d**), ascending pharyngeal artery (**e**), and middle meningeal artery (**f**)

Hemangioblastomas are typically located in the posterior parts of the cerebellum, the spinal cord (Fig. 3), and the medulla oblongata. However, they also occur in the anterior parts and rarely even supratentorially. Cerebellar and spinal hemangioblastomas typically have a strongly enhancing solid and a cystic part. One-third of the larger tumors are purely solid and may have a central necrosis. The strongly enhancing part always has a pial surface, and, depending on its size, flow voids within and around the tumor are visible. Due to the higher protein (erythropoietin) content, the cystic part of a hemangioblastoma is not as hypointense as cerebrospinal fluid on T1-weighted images but strongly hyperintense on FLAIR and T2-weighted images. In contrast to the pilocytic astrocytoma, the wall of the hemangioblastoma cyst does not enhance. The solid and the cystic tumor portions of CNS hemangioblastoma may grow; however, tumor growth is often dominated by increasing cyst size (Fig. 4).Retinal hemangioblastomas (“angiomas”) are best detected by standard ophthalmological funduscopy with dilated pupils. Early detection of small tumors facilitates the treatment by laser coagulation with minimal risk for the vision. Only large tumors are characterized by strongly enhancing irregular structures in the dorsal part of the globe. They can be associated retinal detachment and a V-shaped configuration of the globe especially when they show an exophytic growth towards the subretinal space. Endolymphatic sac tumors (ELST) are usually irregular petrous bone masses, which originate from the endolymphatic epithelium and arise within the intraosseous portion of the endolymphatic duct/sac of the vestibular aqueduct (Fig. 5).

In order to detect tiny tumor nodules and to distinguish them from vessels, high-resolution images are needed (Fig. 2). The Freiburg protocol includes a sagittal T1-weighted contrast-enhanced cranial sequence with fat saturation and 1-mm^3^ isotropic voxels (4:53 min) followed by sagittal T1-weighted 3-mm thick slices of the upper and lower spine (4:45 min each). Axial 3-mm thick slices through suspicious lesions in the spinal canal and T2-weighted sequences may be added.

## Neurosurgical treatment of hemangioblastomas

VHL patients typically develop multiple CNS hemangioblastomas. Still, it is not necessary to surgically remove all of these tumors. Usually a wait-and-scan policy with repeated MRI scans can be recommended in asymptomatic patients.

## Surgical timing

The up to now unpredictable growth behavior of hemangioblastomas makes surgical indication difficult, as these tumors do not necessarily grow continuously. They may be stable in size for an unknown time before the solid tumor part will begin to proliferate. At an uncertain point in time, hemangioblastomas can then stop growing and remain stable again, before proliferating anew later [35]. Previous studies have shown that about half of all CNS hemangioblastomas were stable in size over long-term follow-up [39, 40]. CNS hemangioblastomas may also develop peritumoral edema or, finally, a typical tumor-associated cyst that may rapidly increase in size compressing and damaging surrounding neural tissue [41, 42].

Particularly in pediatric patients with few studies available, the optimal timing of surgical intervention remains a matter of debate [29, 30]. Lifelong repeated MRI scans of the brain and spinal cord (annual) are recommended for VHL patients, irrespective of the number of previously known tumors or absence of symptomatology [4, 28, 29, 42].

There is general consensus that symptomatic tumors should be surgically removed. For asymptomatic but progressive tumors in follow-up MRI, however, treatment strategies differ in the literature. Some authors are hesitant about tumor resection in this situation because the asymptomatic patient is exposed to the surgical risk including neurological damage [31]. In contrast, most authors (including us) advocate surgical resection for growing tumors that do not (yet) cause clinical symptoms, since neurological symptoms that already have developed in the natural course of VHL disease are usually not reversible and hemangioblastoma surgery can be performed with low morbidity in experienced hands [30, 43, 44]. Still, this strategy has to be adapted in patients with repeated previous operations and multiple hemangioblastomas.

Ultimately, this means that it is currently always necessary to work out a tailored and personalized therapy strategy with respect of the location of the tumor, change of tumor size or associated cyst, and symptomatology and general condition of the VHL patient. This can only be sufficiently accomplished in a multidisciplinary team approach in specialized centers involving the patient and parents.

## Principles in hemangioblastoma surgery

Fundamentally, the special aspects of pediatric surgery and anaesthesiological management have to be considered. Advanced perioperative medical care and patient blood management are of utmost importance, especially for young children with low circulating blood volume surgery for highly vascularized tumors such as hemangioblastomas [29, 45]. In this context, a precise microsurgical technique is essential, both to minimize blood loss and to maintain an appropriate anatomical overview.

**Fig. 6:**
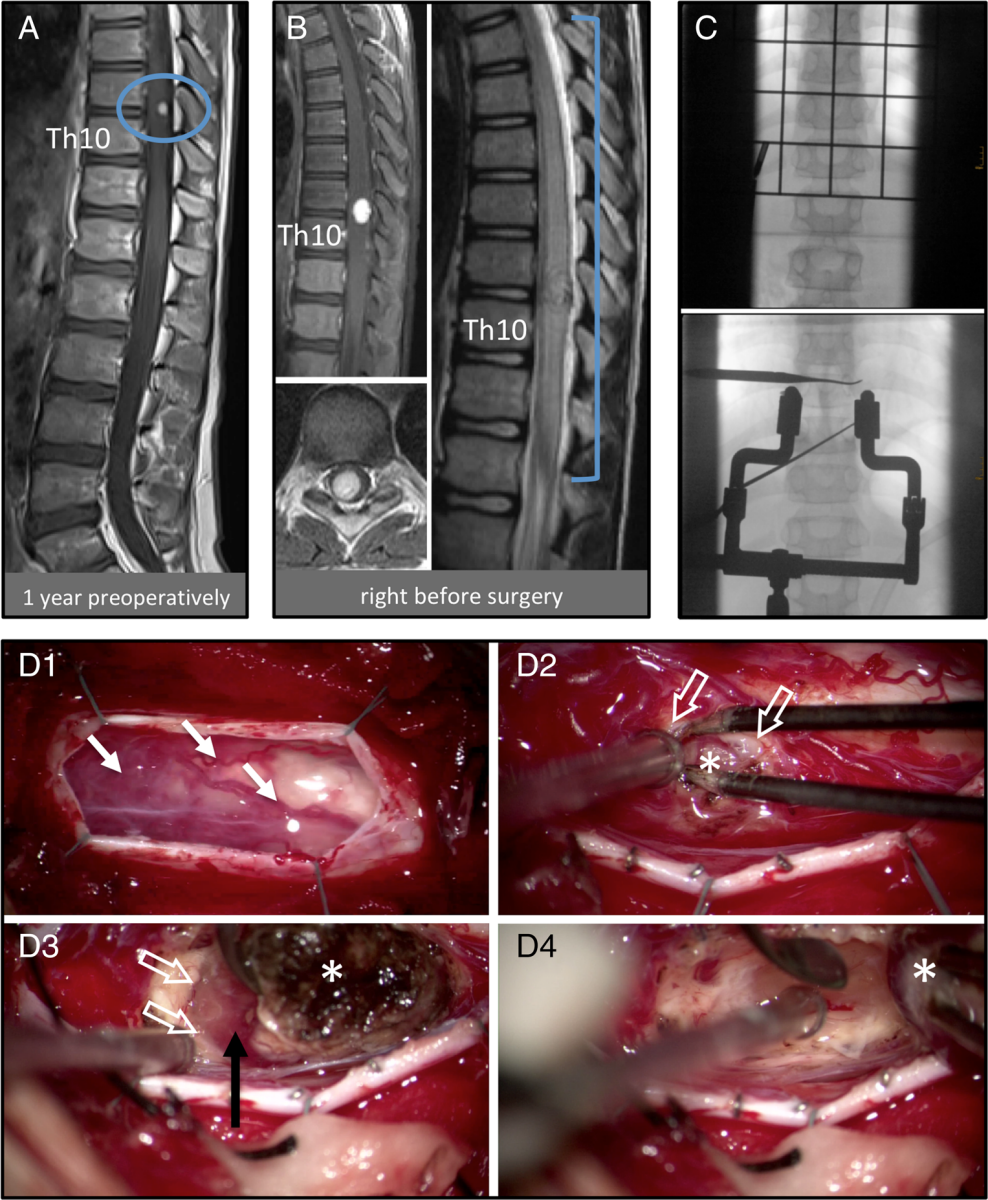
Surgical case illustration. The 10-year-old VHL patient had a previously known small intramedullary hemangioblastoma at Th9-Th10 (encircled in A). Meanwhile the patient developed progressive painful dysesthesia in the right leg. The current MRI (B) showed the solid tumor progressive from 6 × 5 mm to 15 × 10 mm, now with peritumoral myelon edema ranging from Th4 to the conus medullaris. Intraoperative radiographic confirmation of the target level must be as radiation minimized as possible in pediatric patients (here: only 2 single X-ray images taken, note the applied beam collimation). Under microscopic magnification (D1), the dura is opened lengthwise after laminotomy, revealing abundant pathological vessels on the surface of the myelon (arrows). The next crucial step is to identify and incise the junction between the tumor surface and the surrounding neural tissue (D2; asterisk: tumor; hollow arrows: junction). Step by step (D3), the tumor is circumferentially dissected en bloc (asterisk: cauterized tumor surface; black arrow: natural, typically reddish tumor surface; white hollow arrows: plane of dissection between tumor and neural tissue). With this technique (D4), the tumor (asterisk) can be gently dissected from the surrounding neural tissue

The aim of microsurgical treatment is the complete resection of the solid tumor component. Under the surgical microscope/exoscope and using magnification, the typical reddish or orange tumor and tumor-associated blood vessels can be identified. The blood vessels supplying versus draining the tumor need to be identified and then carefully cauterized and transected, vessel by vessel with constant attention to swelling or bleeding of the tumor itself. Next, the junction between the tumor surface and the surrounding neural tissue is identified and incised. This is a crucial step that requires considerable experience in neurosurgical and hemangioblastoma surgery. There is no natural capsule or histologically defined border zone. However, in most instances, the dedicated surgeon is able to find a plane of dissection that facilitates rather bloodless preparation and it is key to constantly control and regain this surgically defined plane of dissection, in which development proceeds only with delicate manipulation. It is very important not to enter the highly vascularized tumor, as this can lead to extensive bleeding. While removing the solid tumor component, the adjacent cyst is usually opened and collapses after resection of the solid part. Arteries bypassing the tumor and feeding eloquent neural tissue have to be spared. In case of doubt, such blood vessels can be temporarily clipped, and only intraoperative neuromonitoring (see below) may reveal whether the vessel can be severed (Fig. 6) [42, 46, 47].

**Fig. 7:**
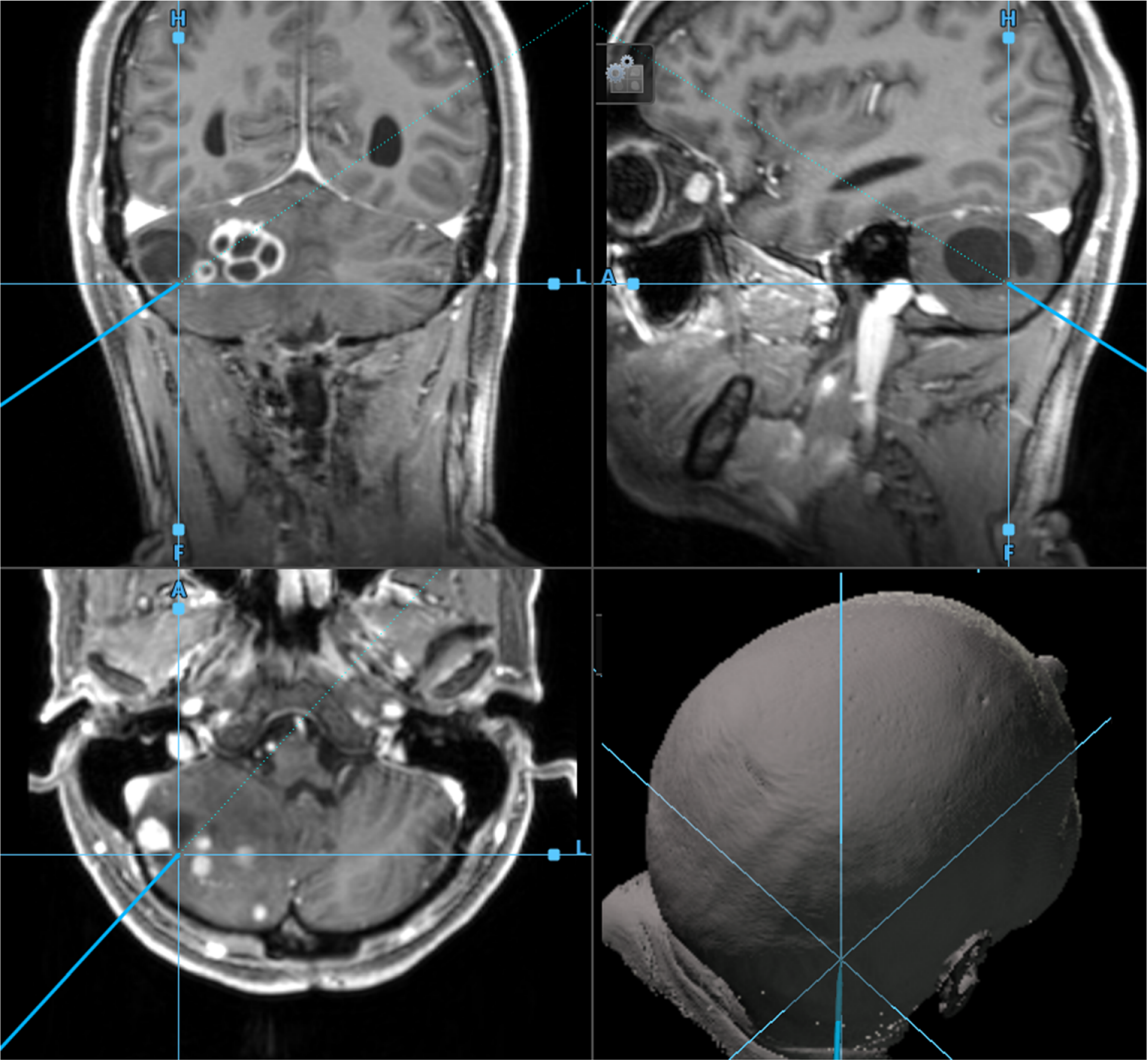
Intraoperative 3D neuronavigation. A preoperatively obtained, high-resolution MRI scan can be used for intraoperative 3D neuronavigation in triplanar visualization. The oblique turquoise line corresponds to a navigation pointer inserted into the posterior fossa, which harbors multiple hemangioblastomas in solid and cystic formation

**Fig. 8:**
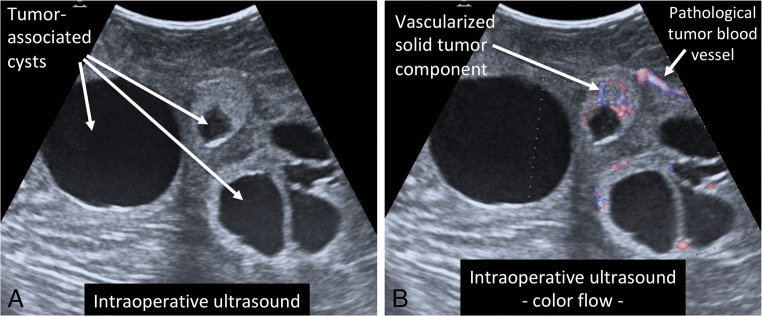
Intraoperative ultrasound. Intraoperative ultrasound showing multiple cerebellar hemangioblastomas with tumor-associated cysts (**a**). The color flow feature (**b**) allows visualization of the blood flow including the high vascularization of the solid tumor component as well as pathological blood vessels supplying the hemangioblastoma

**Fig. 9:**
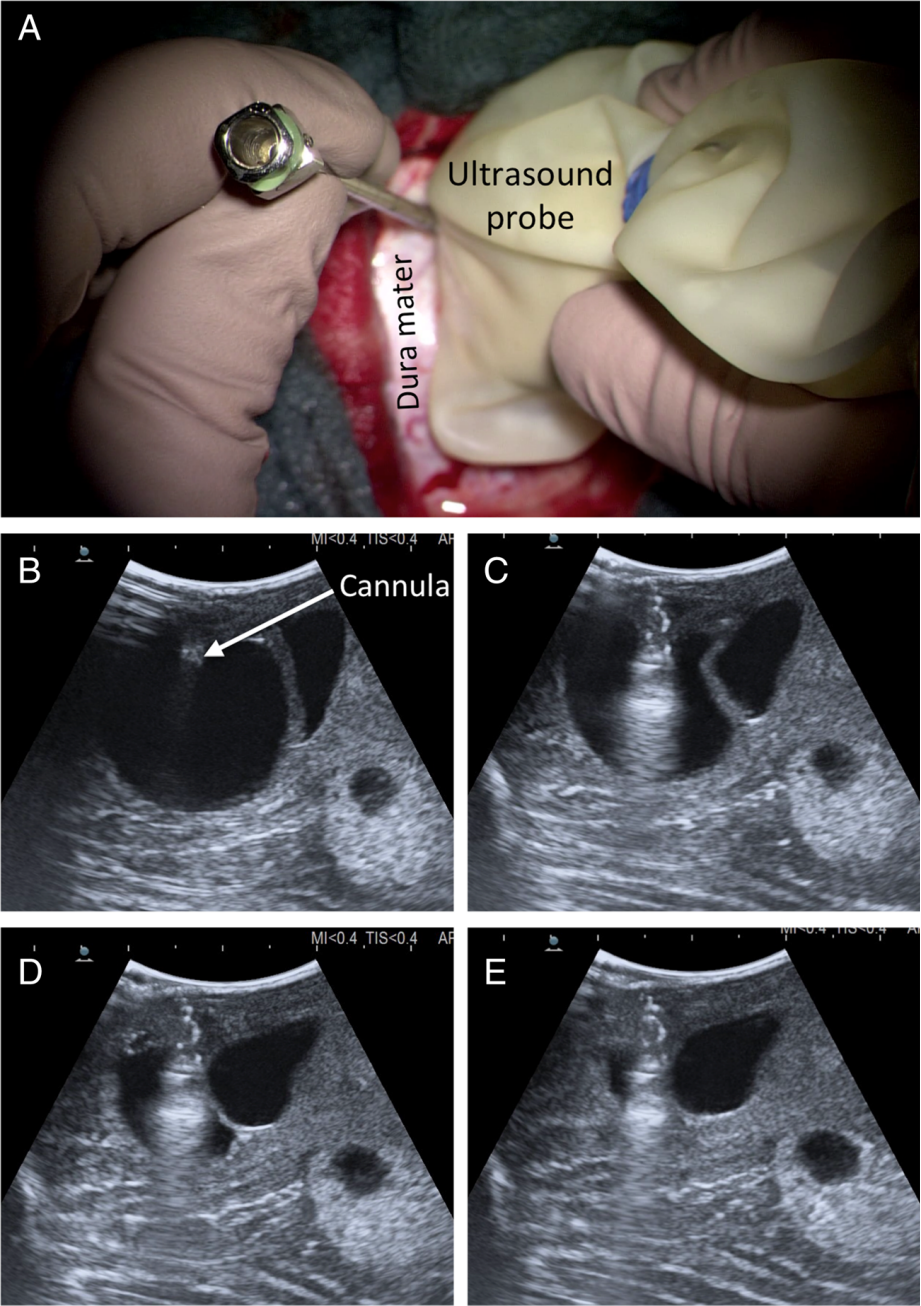
Intraoperative ultrasound-guided cyst evacuation. Intraoperatively, it may be advantageous to first evacuate a large tumor-associated cyst in order to reduce the intraparenchymal pressure. Therefore, after minimal incision of the dura, a cannula is advanced into the cyst under ultrasound guidance (**a**) with sequential outflow of the cyst fluid (**b**–**e**)

Intraoperative 3D neuronavigation (Fig. 7) and intraoperative ultrasound (Figs. 8 and 9) facilitate minimally invasive surgery [48], localization of deeply located tumors, and intraoperative verification of the extent of tumor resection [42, 49].

## State-of-the-art surgical techniques

In recent years, progress in anesthesia and intensive care management as well as technical and surgical advances have led to small but meaningful improvements for pediatric VHL patients.

**Fig. 10:**
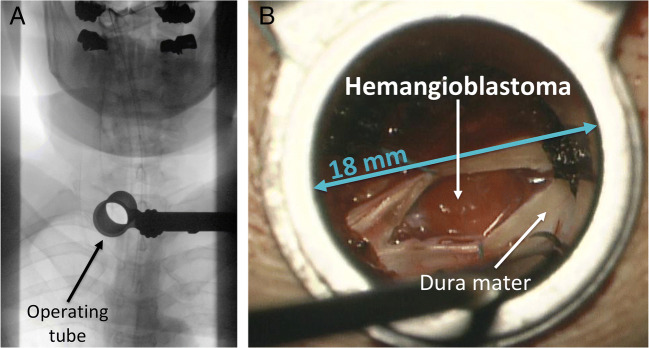
Minimally invasive tumor removal. **a** Intraoperative anterior-posterior X-ray image confirming the installed operating tube at the correct level Th1. **b** Magnified microscopic view through an 18-mm diameter operating tube onto the opened dura mater and the exposed spinal hemangioblastoma at the dorsal root entry zone

We demonstrated in a first series of spinal hemangioblastomas the feasibility and safety of minimally invasive tumor removal via an operating tube (Fig. 10) [47]. Related advantages are less tissue trauma, less blood loss, and shorter hospital stays [47, 50]. Likewise, minimally invasive tubular removal of selected cerebellar hemangioblastomas is possible [48].

**Fig. 11:**
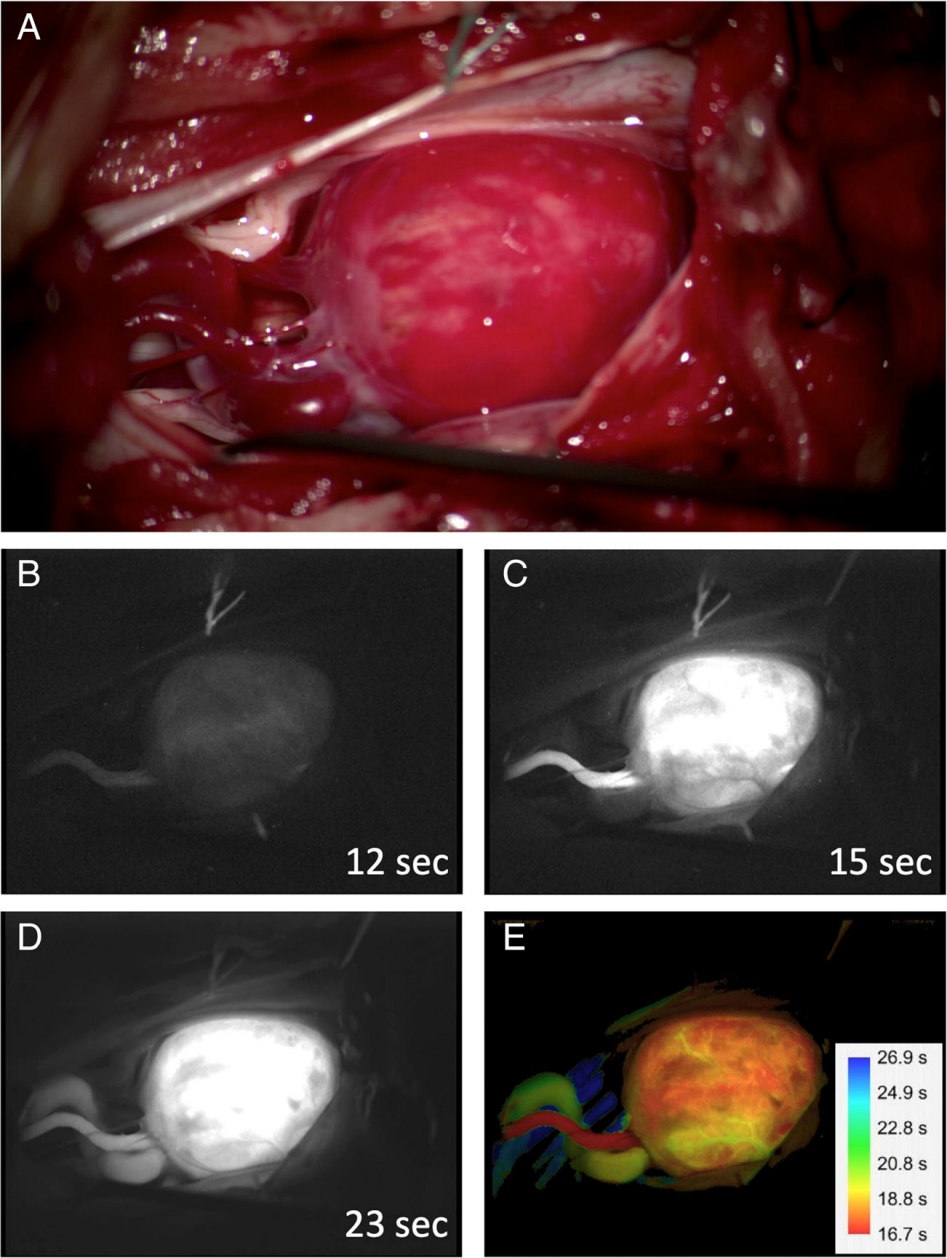
Intraoperative indocyanine green (ICG) videoangiography. The microscope image (**a**) shows a spinal hemangioblastoma and indistinguishable pathological blood vessels. Especially in spinal hemangioblastomas, it is important to cauterize and sever the feeding vessels first, and not the draining vessels. Otherwise, swelling or even hemorrhage out of the tumor might occur. Intraoperative videoangiography with snapshots at 12, 15, and 23 s after ICG injection (**b**, **c**, **d**) allows the identification of a thin feeding vessel after 12 s (**b**) and major draining vessel after 23 s (**d**). Post-processing color coding according to the injection time indicates the feeding vessel in red and the major draining vessel in yellow/green (**e**)

Furthermore, fully microscope-integrated intraoperative videoangiography with the fluorescent dye indocyanine green (ICG) can be used to distinguish between feeding and draining vessels (Fig. 11) [51].

Intraoperative neuromonitoring is a further safety tool to monitor nerve function during surgery [42, 52]. Neuromonitoring is especially helpful for tumors with relation to the cranial nerves, the brainstem, or the fourth ventricle to enable the surgeon for adjusting the surgical strategy. In intramedullary hemangioblastoma, a stimulation probe can be used to identify the corticospinal tract and dorsal column to facilitate transmedullary access to ventrally located hemangioblastomas and to avoid substantial motor deficits [52, 53].

**Fig. 12:**
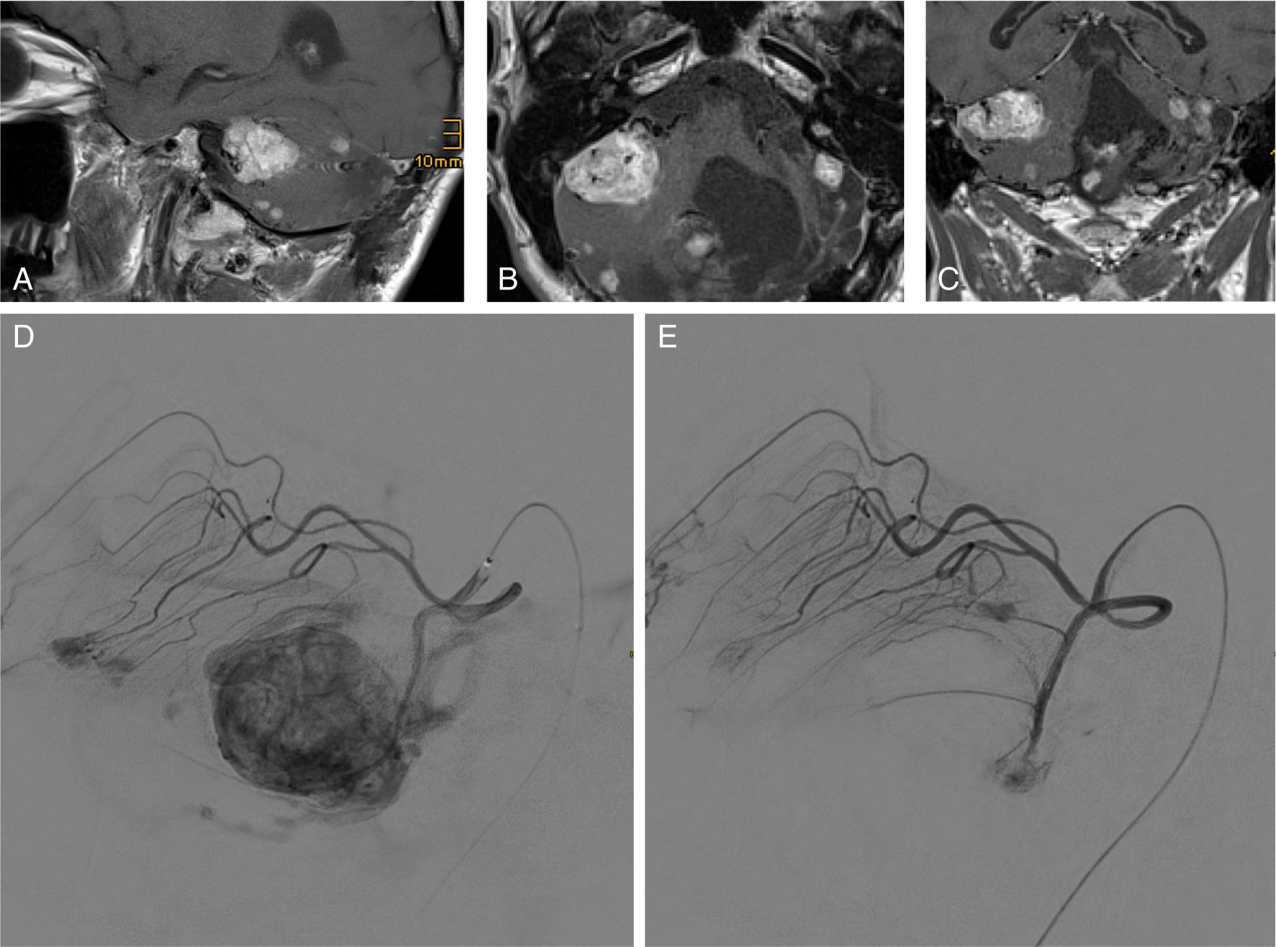
Preoperative embolization. Triplanar contrast-enhanced, T1-weighted MRI scan (**a**, **b**, **c**) predominantly showing a large solid tumor of the right posterior fossa in a VHL patient. Notice the large pathological blood vessels in and around the tumor (black) (**b**). Preoperative angiography (**d**) depicting a large tumor blush of the hemangioblastoma that is mainly nourished from the superior cerebellar artery. After particle embolization (**e**), the blood supply has been completely eliminated

Large solid hemangioblastomas harbor a considerable risk of intra- and postoperative hemorrhage [37]. Most authors recommend preoperative embolization in these cases to minimize bleeding tendency (Fig. 12) [42]. Since embolization can involve severe complications in individual cases [42, 54], potential benefits and risks must be considered. In addition, endovascular treatment should be reserved for specialized centers with extensive expertise.

## Alternative treatment of CNS hemangioblastoma

Stereotactic radiosurgery has been recently proposed as safe and effective treatment modality for stabilizing the tumor size of CNS hemangioblastomas in adults, particularly in cases of multiple or recurrent tumors and patients who are not surgical candidates [55–57]. This presumed effect of radiosurgery however can also be attributed to a coincidentally simultaneous quiet phase of tumor growth. Therefore, radiosurgery should only be considered in an ultima ratio situation in which CNS hemangioblastomas cannot be controlled surgically and indeed require treatment. It should also not be applied prophylactically for multiple tumors. Moreover, there is little data on stereotactic radiosurgery and its late complications in children [29].

Medical therapy is at an experimental stage. To date, no chemotherapy has proven effective on hemangioblastoma growth.

## Prognosis and aftercare

Several studies illustrate favorable outcome after surgical removal of hemangioblastoma in the cerebellum, spinal cord, and brainstem [31]. The resection of cerebellar lesions upon presence of symptoms in VHL patients resulted in improvement or stabilization in 98% of cases [46]. Long-term imaging follow-up at 5 years did not reveal recurrence. Spinal cord hemangioblastomas can also be safely resected, and complete surgical resection is curative [58]. Ventral tumors or completely intramedullary tumors might be associated with an increased risk of postoperative worsening. Complete resection of symptomatic lesions led to neurological stability in 96% of cases. After 5 and 10 years, 86% and 78% of patients remained functionally stable indicating that the long-term deterioration of neurological function is usually associated with the progression of VHL disease [58].

Asymptomatic CNS hemangioblastoma may enter a phase of stable disease for many years and show favorable course without any treatment and define need to adapt follow-up and screening examinations [35]. On the other hand in VHL patients, CNS hemangioblastomas remain the leading cause of death next to renal cell carcinoma [59]. For VHL patients, tumor burden at diagnosis and young age seem to be the most important risk factors for new tumor development [35]. Prognosis further improved for VHL patients due to consistent use of surveillance programs with early detection of associated tumors [59]. Experts from different countries proposed recommendations for genetic counseling and molecular genetic screening for germline mutations of the *VHL* gene and clinical investigations. These, however, vary regarding the first age for the target organs and the intervals and the methods of clinical investigations. This is also the case of the authors here, but most of us agree with these recommendations. Molecular genetic testing should be performed:

- In every patient with newly diagnosed CNS hemangioblastoma,
- With newly diagnosed VHL-typical disease manifestations of the eyes or visceral organs, or
- With a positive family history for VHL-associated tumors.

Carriers of *VHL* germline mutations should immediately undergo complete clinical investigations according to the proposed screening program.

## Prevention medicine in VHL disease

Morbidity and mortality in VHL patients have decreased substantially due to better insight into the pathophysiology and natural history of the clinical manifestations, better imaging techniques, and improvements in therapy. Careful surveillance is critical to early detect lesions and monitor asymptomatic manifestations for progression with the aim to prevent severe sequelae such as persistent neurological deficit, blindness, and death.

The optimal frequency for various imaging and screening procedures, which attempts to balance the risks and costs versus the potential for a delayed diagnosis, is not known. Available surveillance recommendations for pediatric VHL patients are inhomogeneous and mainly based on small case series and expert opinions. Regarding CNS hemangioblastoma, the Danish recommendations include one baseline MRI of the CNS between 5 and 14 years of age and then follow-up MRI every 1–2 years from the age of 15 years. The Dutch protocol, the American VHL alliance, and the Clinical Cancer Research (CCR) pediatric oncology series recommend biennial MRI scans starting at age 15, 16, and 8 years, respectively [17, 28]. Yet other authors advocate annual MRI scans from the age of 11 years [4]. British guidelines propose every 12–36 months in adolescence [28]. The heterogeneity of these recommendations therefore does not permit clear and reliable guidelines.

The Freiburg VHL screening program covers approximately 300 MRIs of the CNS of monitored VHL patients each year, with about 15 of these being pediatric patients. Our experience from 30 years Freiburg VHL Registry with approximately 600 operations on CNS hemangioblastomas shows that pediatric VHL patients become very rarely symptomatic from CNS hemangioblastoma. The youngest symptomatic patient in our series was 10 years old. In this patient, the arising symptoms could not be anticipated despite an MRI 1 year before. The youngest VHL patient in our series who underwent surgery for an asymptomatic CNS hemangioblastoma, which had grown during the surveillance period, was 14 years old.

This substantial expertise in surveillance and neurosurgical treatment over the last 30 years has resulted in the following Freiburg screening protocol for VHL patients including relatives who share the given *VHL* germline mutation (Table 1):

- In asymptomatic VHL patients: first contrast-enhanced MRI of the CNS at the age of 14 years, thereafter biennial MRI until the age of 18 years, then annual MRI surveillance as in adults;
- In asymptomatic VHL patients: in case of major pathologies in MRI of the CNS, the follow-up MRI interval should be shortened according to an individual assessment;
- In VHL patients who have become symptomatic: immediate MRI of the CNS, independent of age.
- Screening for retinal angiomas should be started at 6 years.
- Screening for pheochromocytomas should be started at age 6 by plasma metanephrines biannually and in patients with hypertension, sweating, and palpitations by MRI of the abdomen.
- Screening for kidney and pancreas tumors should be started at age 12 by MRI of the abdomen.

**Table 1:** Freiburg screening protocol for children with diagnosed VHL disease

Tumor	Recommended surveillance	Start age	Frequency
Retinal hemangioblastoma	Retinoscopy after dilatation	6 years	Biennial
Pheochromocytoma and paraganglioma	Blood pressure at all medical visits	6 years	
	Plasma fractionated metanephrines or 24 h urine fractionated metanephrines	6 years	Annual
Endolymphatic sac tumor (ELST)	Audiogram	6 years	Biennial
CNS hemangioblastoma	MRI brain and spine +/− contrast	14 years	Biennial
Renal cell carcinoma	MRI abdomen	12 years	Annual
Pancreatic neuroendocrine tumor	MRI abdomen	12 years	Annual

For progressive or critical tumors, the examination interval should be adjusted and accordingly shortened

Experiences made by many patients in Germany, but also in many other countries, show that the waiting time from asking for a clinical investigation date is a complex procedure; currently the intervals last mostly several months and coordination of investigations for personal interview by the coordinator, retinoscopy, CNS-MRI, and MRI of the abdomen for 1 day is a challenge. One must, however, take in account that these patients have already the psychological burden of knowing of a lifelong very high risk for tumors. Once patients have decided to undergo a new investigation, many of them can experience psychological alterations. Therefore, intervals between receiving the request for clinical investigations and the offered date should be as short as possible, meaning less than a month. Reporting of the investigation results and their interpretation as well as the decisions, the VHL board should be communicated to the patients and families in a coordinated and timely fashion. In our experience, every day counts for the patients, and all this should not last more than a week.

Organized VHL monitoring of patients aims to minimize the burden on families, increase surveillance compliance, and allow early detection of manifestations and thus optimized timing of surgery. Since such tumor surveillance is time-consuming and requires financial and psychosocial efforts, screening programs should be run by experienced, multidisciplinary teams coordinated by age-adequate providers.

## Pediatric challenges

Regarding the pediatric population, CNS hemangioblastoma in VHL disease challenges health care providers in several ways. Diagnosis of CNS hemangioblastoma or multiple retinal hemangioblastoma in childhood nearly always leads to diagnosis of VHL disease. Although VHL-associated tumors may manifest in early childhood, detection of e.g., high blood pressure and vision loss in infants or even paresthesia in teenagers remains a major problem in pediatric reality. Improved education of caregivers and providers is needed to avoid delayed diagnosis. Childhood hemangioblastomas in the posterior fossa can be dramatic events due to delayed diagnosis and peritumoral cysts with the potential of rapid size increase.

Regarding outcome of surgical therapy, the majority of symptomatic children showed improved clinical symptoms after surgical removal of CNS hemangioblastoma in experienced hands (72.2% improved, 19.4% remained stable), with postoperative complications being generally minor and temporary [29, 30].

## Conclusion

Identification of CNS hemangioblastomas, other disease manifestations typical of VHL, or a positive family history should lead to screening for germline mutations of the *VHL* gene. Every VHL-affected child should be offered to attend a multidisciplinary screening program. If removal of CNS hemangioblastomas is indicated, this can be performed safely in specialized centers using modern surgical techniques.
